# Identification of dominant global and local modes behind dynamic stiffness valleys in BIW structures via modal contribution and ESE

**DOI:** 10.1371/journal.pone.0334932

**Published:** 2025-10-16

**Authors:** Mingsheng Wang, Wendi Wang, Tao Chen, Xiangwei Lu, Xuelian Yi

**Affiliations:** 1 Dazhou Vocational and Technical College, Sichuan, China; 2 SERES Automobile Co., Ltd., Chongqing, China; Ural Federal University named after the first President of Russia B N Yeltsin Institute of Physics and Technology: Ural'skij federal'nyj universitet imeni pervogo Prezidenta Rossii B N El'cina Fiziko-tehnologiceskij institut, RUSSIAN FEDERATION

## Abstract

This study proposes a novel methodology that integrates modal contribution analysis with Element Strain Energy (ESE) distribution to identify the dominant modes causing dynamic stiffness variations in Body-in-White (BIW) structures. As Noise, Vibration, and Harshness (NVH) performance becomes increasingly critical in automotive design, accurately identifying the sources of dynamic stiffness deficiencies in the early design stages is imperative. This research addresses a significant gap in existing literature, where traditional methods struggle to distinguish between global and local modes in high-frequency, dense modal environments. By systematically analyzing the impact of both global and local modes on dynamic stiffness at key vehicle body connection points, our findings demonstrate the critical importance of prioritizing higher-frequency modes with localized strain energy concentrations for early-stage structural analysis. The proposed approach effectively tackles challenges such as modal overlap and frequency discrepancies, thereby enhancing the precision of NVH diagnostics and providing a reliable framework for targeted structural optimization. Consequently, this work offers a substantial advancement in the understanding and enhancement of NVH performance in automotive body structures, contributing to more efficient and effective design processes.

## Introduction

Noise, Vibration, and Harshness (NVH) performance is a primary metric for evaluating vehicle quality and passenger comfort in modern automotive design, serving as an essential industry benchmark. Driven by technological advancements and heightened consumer expectations, superior NVH performance—encapsulating the occupant’s subjective experience of refinement—is now a decisive factor for brand competitiveness [1,2].

The Body-in-White (BIW) forms the structural core of a vehicle, fulfilling critical functions beyond supporting the powertrain, chassis, and battery systems by directly governing the transmission paths of structural vibrations and airborne noise. Consequently, the NVH properties of the BIW are fundamental to the overall acoustic and vibrational behavior of the vehicle [3,4]. Most passenger cars utilize a unibody structure; however, the specific stiffness distribution inherent to this design often couples low-frequency vibrational energy into the cabin, making it a primary source of structure-borne noise [5–8].

The Finite Element Method (FEM) is an established virtual simulation tool, indispensable for the early-stage prediction and optimization of NVH behavior through dynamic performance assessment [9–11]. Within the FEM framework, modal analysis constitutes a fundamental technique for efficiently elucidating a structure’s inherent dynamic characteristics [12,13]. Complementing modal analysis, the Element Strain Energy (ESE) method identifies regions of high strain energy concentration, establishing it as a critical criterion for pinpointing local stiffness deficiencies [14,15]. A principal objective in vibration control is the identification and reinforcement of these structurally weak regions to mitigate undesirable responses. Consequently, ESE-based analysis provides a powerful mechanistic foundation for guiding targeted structural optimization [16–20].

In optimizing the NVH performance of the BIW, dynamic stiffness represents a critical parameter for evaluating the structure’s resistance to dynamic forces and identifying local weaknesses. This property is intrinsically linked to modal characteristics, including natural frequencies, mode shapes, and modal participation factors [15,21–26]. Recent research has advanced various methodologies for BIW stiffness enhancement: multi-objective optimization and design of experiments have been employed to improve bending and torsional stiffness at critical structural points [27]; parametric studies have explored variations in global static and dynamic stiffness characteristics [28]; and investigations into stiffness-vehicle dynamics interactions have provided insights for comprehensive optimization [29]. Furthermore, topology and thickness optimization techniques have been developed to enhance dynamic stiffness under lightweight constraints [30,31], while specialized evaluation criteria for local stiffness have been established to support NVH and handling refinement [32,33]. These collective efforts highlight the importance of integrated vibration and structural optimization in BIW development

Despite significant advances in enhancing the dynamic stiffness of BIW structures, a critical challenge remains in accurately identifying the root causes of stiffness deficiencies. The interaction between global and local modes in high-frequency regimes presents a particularly complex problem, exacerbated by increasing modal density and overlap. BIW modal behavior can be categorized into global modes—such as bending or torsional deformations that induce large-scale structural motion—and local modes, which are confined to specific regions or joints. While global modes generally cause broad reductions in dynamic stiffness across multiple connection points and elevate low-frequency noise transmission, local modes may lead to sharp stiffness reductions in narrow frequency bands near critical locations. Existing research has predominantly focused on mitigating global modes through enhancements to structural continuity and stiffness along primary load paths. However, the accurate identification of the dominant modal mechanism—global or local—underlying a specific dynamic stiffness valley is essential for effective structural optimization.

To address this gap, we introduce a novel methodology that systematically differentiates the contributions of global and local modes to dynamic stiffness deficiencies in BIW structures. Unlike conventional approaches that treat the structure monolithically, our technique integrates modal contribution analysis with ESE distribution to precisely classify modal influences, even in high-density modal environments with significant overlap and frequency discrepancies. This distinction provides a rigorous foundation for targeted structural modifications, enabling more effective NVH performance improvements during early design stages.

## Theoretical bases

For illustration, the dynamic behavior of a single-degree-of-freedom (SDOF) system is described by the following governing equation:


$$\begin{array}{c} \text{m}\ddot{x}+c\dot{x}+kx=f \end{array}$$ (1)

where *f* is the excitation force; m is the mass; c is the viscous damping coefficient; k is the stiffness; and *x* is the displacement.


$$\begin{array}{c} x=Xe^{j(\omega t-\varphi )} \end{array}$$ (2)

$$\begin{array}{c} \dot{x}=j\omega Xe^{j(\omega t-\varphi )} \end{array}$$ (3)

$$\begin{array}{c} \ddot{x}=-\omega^{\text{2}}Xe^{j(\omega t-\varphi )} \end{array}$$ (4)

$$\begin{array}{c} f=Fe^{j\omega t} \end{array}$$ (5)

The Dynamic Stiffness, $K_{d}(\omega )$, characterizes the frequency-dependent relationship between an applied harmonic force and the resulting displacement at a given excitation point. It is a key metric for evaluating a structure’s ability to resist dynamic loads, particularly at critical connection points. It is defined as:


$$\begin{array}{c} K_{d}(\omega )=\frac{F(\omega )}{X(\omega )} \end{array}$$ (6)

When an external force acts on an elastic structure, the mechanical work is partially stored as strain energy. The total strain energy, incorporating both normal and shear strains, is expressed as:


$$\begin{array}{c} E=\frac{\text{1}}{\text{2}}\int_{\varepsilon }\left(\sigma_{x}\delta_{x}+\sigma_{y}\delta_{y}+\sigma_{z}\delta_{z}+\tau_{xy}\eta_{xy}+\tau_{xz}\eta_{xz}+\tau_{yz}\eta_{yz}\right)d\varepsilon \end{array}$$ (7)

Here, σ and τ represent the normal and shear stresses, while δ and η are the corresponding strains; ε is the integration domain.

In finite element analysis, strain energy can be formulated in matrix form using the mode shape vector and stiffness matrix:


$$\begin{array}{c} E=\mu_{n}^{T}k\mu_{n} \end{array}$$ (8)

where $\mu_{n}$ is the mode shape vector of the *n*-th mode, and K is the global stiffness matrix.

Using modal superposition, the physical response of an N-degree-of-freedom system can be expressed as the sum of individual modal responses:


$$x(\omega )=\varphi_{l\text{1}}q_{\text{1}}(\omega )+\varphi_{l\text{2}}q_{\text{2}}(\omega )+\cdots +\varphi_{lN}q_{N}(\omega )=\sum_{r=\text{1}}^{N}\varphi_{lr}q_{r}(\omega )$$ (9)

where $\varphi_{lr}$ is the modal coefficient at the *l*-th point for the r-th mode; $q_{r}(\omega )$ is the corresponding modal coordinate, and N is total number of modes. The modal response of the *r*-th mode is:


$$\begin{array}{c} x_{r}(\omega )=\phi_{r}q_{r}(\omega )=\frac{\phi_{r}F_{r}(\omega )}{-m_{r}\omega^{\text{2}}+j\omega c_{r}+k_{r}} \end{array}$$ (10)

Accordingly, the modal contribution ratio of the r-th mode is defined as:


$$\begin{array}{c} Q_{r}=\frac{x_{r}(\omega )}{x(\omega )}=\frac{\phi_{r}q_{r}(\omega )}{\sum_{r=\text{1}}^{N}x_{r}(\omega )}\times \text{1}\text{0}\text{0}\% \end{array}$$ (11)

## Finite element modeling and analysis setup

The dynamic stiffness assessment in this study was conducted using a detailed finite element (FE) model of the Body-in-White (BIW). The entire simulation workflow was managed within the Altair HyperWorks 2021.1 software environment. The FE model was pre-processed and built using Altair HyperMesh 2021.1. The frequency response analysis was solved using Altair Compute Console 2021.1, and all post-processing tasks, including the interpretation of dynamic stiffness results, mode shapes, and strain energy distributions, were performed in Altair HyperView 2021.1.

The BIW model was simulated under free-free boundary conditions, a standard approach for evaluating the intrinsic dynamic characteristics of a structure without the influence of external constraints. The dynamic stiffness was evaluated through a frequency response analysis. A unit harmonic load (1 N) was applied in the vertical (Z-) direction at each of the key connection points sequentially. The analysis spanned an excitation frequency range of 20 Hz to 500 Hz to cover the NVH region of interest. For each excitation frequency, the response was calculated over a frequency range extending to 1.5 times the excitation frequency to ensure the accurate capture of potential resonant peaks. The structural damping ratio was specified as 3% [34].

## Global vs local modes in Z-direction stiffness of BIW structures

**Fig 1:**
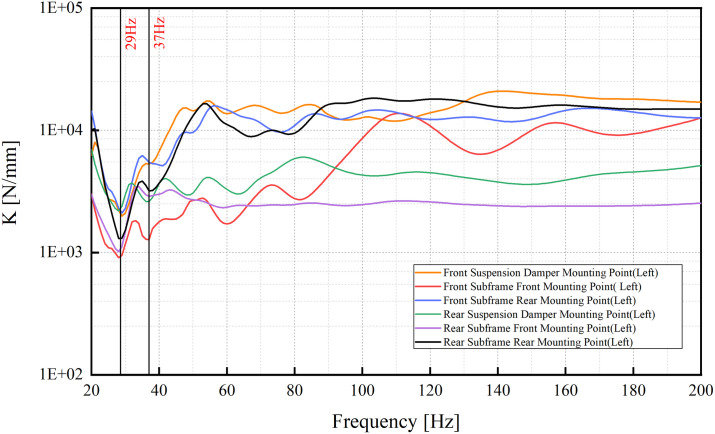
Z-direction dynamic stiffness curves at typical BIW mounting points.

The vertical (Z-direction) dynamic stiffness of the BIW is a critical determinant of vehicle NVH performance, as it directly governs the transmission of noise and vibration from major excitation sources—such as road irregularities, suspension impacts, and powertrain forces—into the cabin. These low-frequency excitations predominantly manifest as audible noise, seat vibration, and structural resonances, critically affecting ride comfort. This study evaluates the Z-direction dynamic stiffness at six key mounting points on the BIW (Fig 1) to assess the structure’s response to these operational loads.

**Fig 2:**
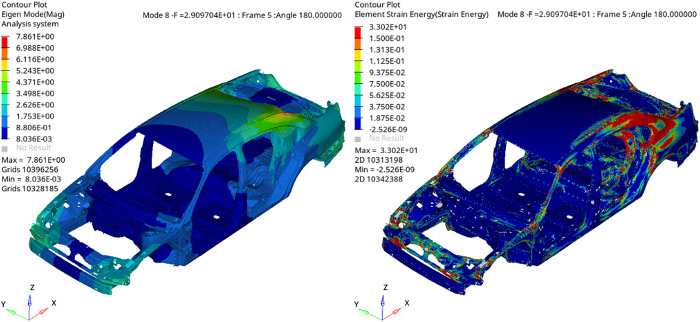
First torsional mode of the BIW and its corresponding ESE distribution.

**Fig 3:**
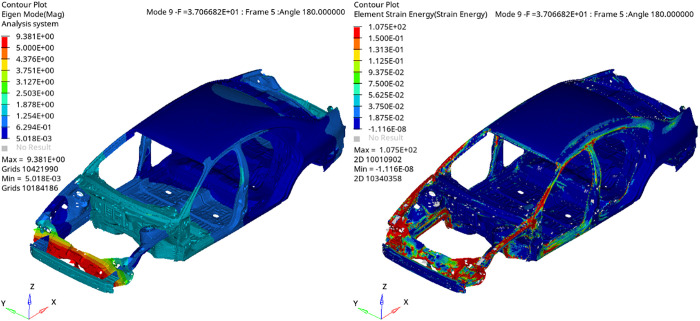
First bending mode of the BIW and its corresponding ESE distribution.

A significant reduction in stiffness was detected at approximately 29 Hz and 37 Hz across all mounting points, indicating a global structural behavior. Modal analysis verified that these stiffness drops correspond to the BIW’s first torsional and first bending modes, respectively (see Figs 2 and 3). These modes involve a collective response of the vehicle body.

In vehicle body dynamics, it is essential to distinguish between global and local modes, as they exhibit different response behaviors and physical properties. while the total strain energy for a given mode n is a scalar quantity (Equation 8), the spatial distribution of ESE across the finite element mesh is of critical diagnostic value. Global modes, exemplified by the first bending and torsional modes, exhibit coordinated deformation along primary structural paths, accompanied by a widespread distribution of ESE. This uniform energy pattern signifies a system-wide response, offering valuable indicators of global dynamic behavior but limiting sensitivity to localized stiffness issues.

**Fig 4:**
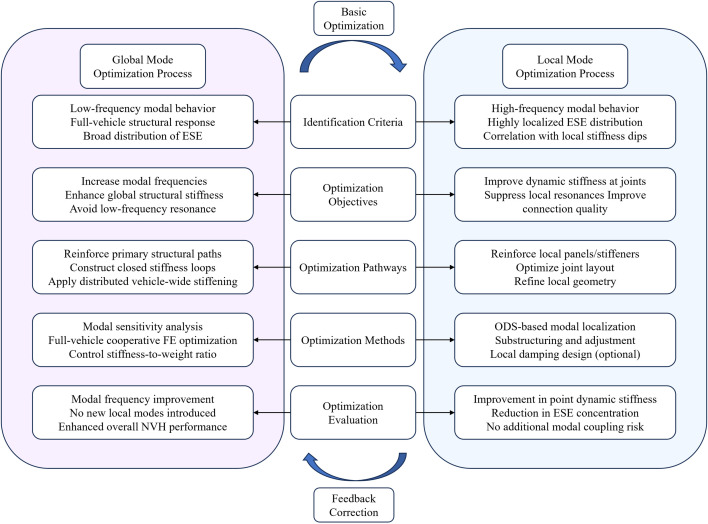
Comparison of global and local modes and their optimization.

Local modes, conversely, are restricted to specific joints or segments, displaying spatially concentrated ESE profiles. This focused energy concentration makes local modes highly effective for identifying precise structural deficiencies and guiding localized mesh refinement or reinforcement. Therefore, although global modes define the fundamental dynamic characteristics, a focus on higher-frequency local modes—with their distinct localized energy signatures—is crucial during early-stage development for targeted optimization of weak areas. Fig 4 summarizes the optimization workflows for the two modal types, highlighting their distinct analysis and design approaches.

## Mode identification based on modal contribution and ESE distribution

**Fig 5:**
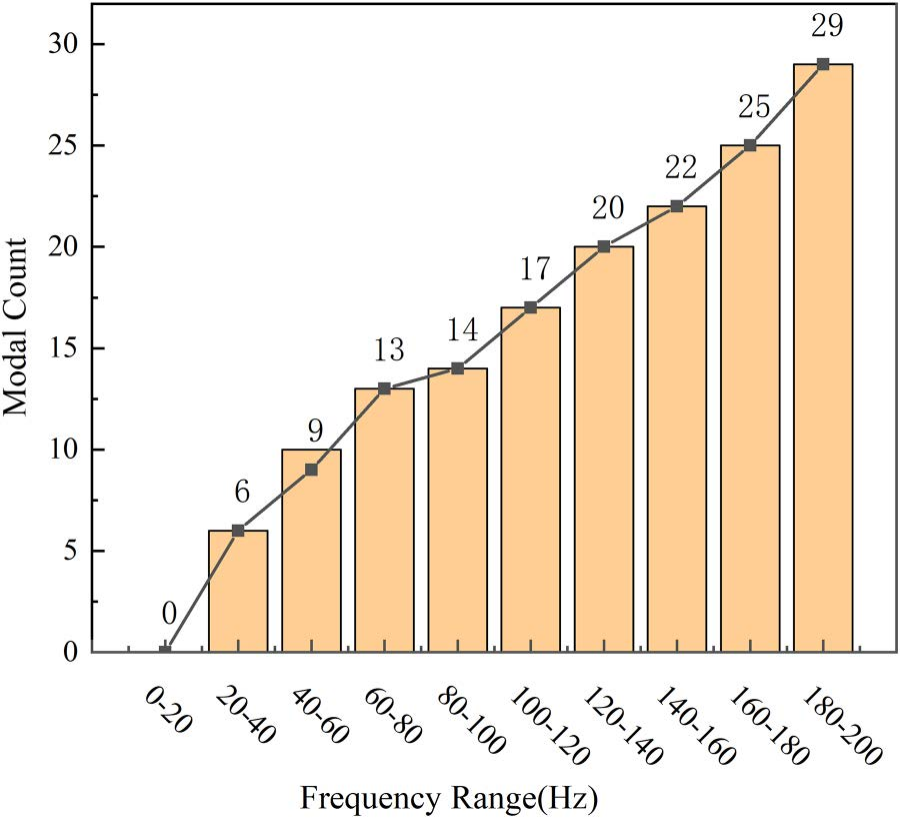
Variation in the number of modes with frequency.

As shown in Fig 5, the number of identifiable structural modes exhibits a substantial increase with frequency, following an approximately linear trend. Particularly above 100 Hz in the mid-to-high frequency range, the mode density rises sharply, leading to reduced frequency spacing between adjacent modes and intensifying modal coupling. This dense modal distribution complicates mode identification, making traditional approaches based on mode shape observation or frequency position prone to misidentification or omission of critical modes.

The modal superposition principle (Equation 9) states that the total physical response at any point is a linear combination of individual modal responses. Under these conditions, relying solely on mode frequency or shape intuition for response attribution is vulnerable to interference from mode overlap and mixed contributions. To address this, modal contribution analysis (Equation 11) is introduced to quantitatively evaluate each mode’s actual participation in the structural response, enabling identification of dominant contributors among numerous modes. Coupled with spatial response patterns, this method further distinguishes global from local modes. This approach is particularly effective in high modal density scenarios, significantly enhancing the accuracy and efficiency of NVH diagnostics and optimization.

The front suspension mounting point (left side) is taken as an example.

**Fig 6:**
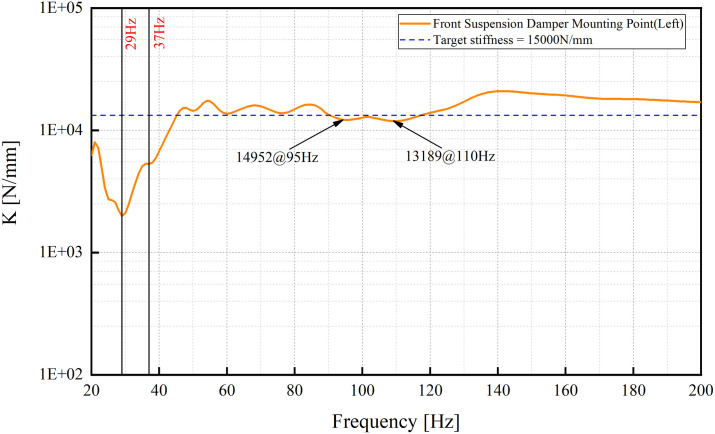
Dynamic stiffness curve.

**Fig 7:**
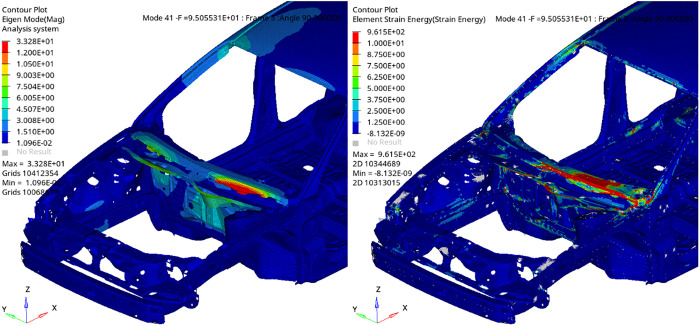
Mode shape and ESE at 95 Hz.

Fig 6 reveals two frequency regions exhibiting insufficient dynamic stiffness. While the low-frequency deficiency is clearly attributable to a local mode, as confirmed by mode shape and ESE distributions shown in Fig 7, identifying the source of the stiffness dip near 110 Hz is more challenging. These challenges arise from two primary factors: the high modal density above 100 Hz (Fig 5) results in closely spaced modes with significant coupling and overlap, complicating conventional modal identification methods. Additionally, discrepancies between the frequency ranges used for dynamic stiffness calculation and modal solution cause misalignment between the observed stiffness valley and individual modal frequencies. As summarized in Table 1, multiple modes are clustered around 110 Hz, making it difficult to pinpoint the dominant contributor through conventional approaches.

**Table 1:** Calculation results of BIW modal section.

Mode	Frequency/Hz	Oscillation mode
49	107.91	Local Modes
50	108.56	Cabin front crossbeam mode
51	110.81	Local Modes
52	111.48	Local Modes
53	112.26	Cabin global mode
54	114.48	Local Modes
55	116.26	Local Modes
56	116.58	Local Modes

To address this challenge, a modal contribution analysis was conducted at 110 Hz to quantify the contribution of each mode. By integrating modal contributions with spatial ESE distributions, the dominant mode type responsible for the dynamic stiffness dip can be effectively identified.

**Fig 8:**
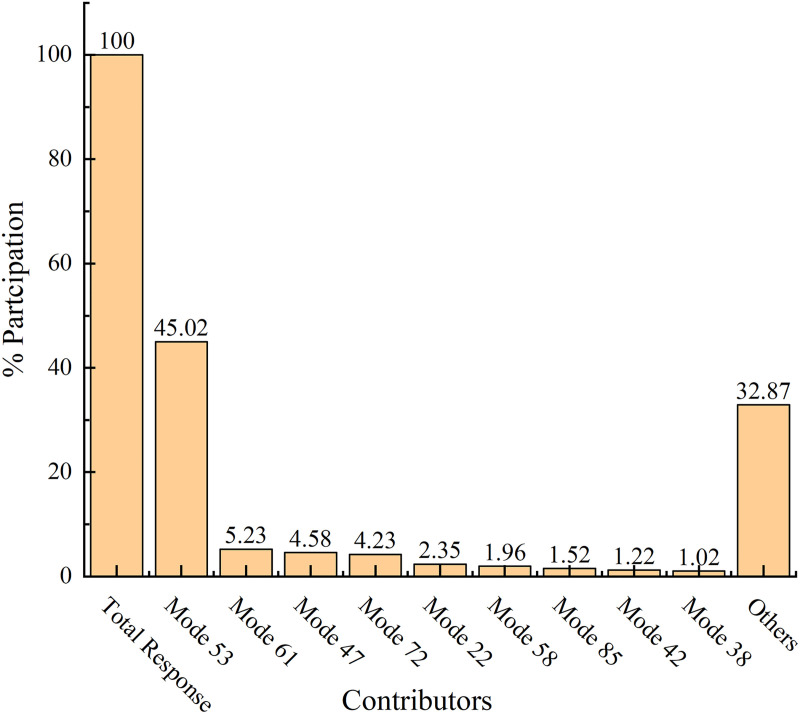
Modal contributions at 110 Hz.

**Fig 9:**
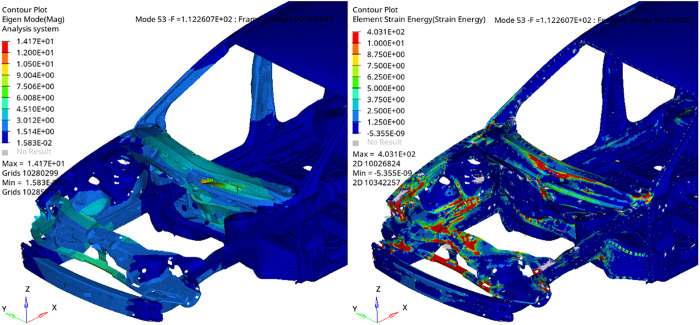
Mode shape and ESE of BIW 53th order mode.

Modal contribution analysis identifies Mode 53 as the dominant contributor to the dynamic stiffness reduction near 110 Hz at the left front suspension mount (Fig 8). The mode shape and ESE distribution (Fig 9) reveal a global, coordinated deformation primarily in the Z-direction, engaging key engine-bay structures such as the front longitudinal members and shock towers. Strain energy is concentrated along critical load paths, including the mid-left rail and subframe connection zone, confirming the global nature of this mode. Although its frequency placing it in the mid-to-high range, the extensive structural involvement and continuous energy distribution of Mode 53 classify it as a global engine-bay mode. Consequently, structural enhancements should target improving stiffness and path continuity within this region.

## Conclusion

This study introduces a unified framework that integrates modal contribution analysis with ESE distribution to distinguish between global and local modes in BIW structures, elucidating their distinct effects on dynamic stiffness. The main findings are as follows: Low-frequency reductions in dynamic stiffness are primarily governed by global modes, which involve coordinated deformation across large structural areas and require improvements to the integrity of global load paths. In the mid-to-high frequency range, dynamic stiffness deficiencies are often dominated by local modes with concentrated strain energy, necessitating targeted reinforcement; however, global modes may still contribute to stiffness reductions in these frequency regimes. The proposed methodology effectively addresses attribution challenges in high modal density environments by quantitatively linking dynamic responses to spatial energy distributions, thereby significantly enhancing diagnostic precision for NVH optimization. This approach provides a robust foundation for improving ride comfort and structural durability. Future work will focus on developing automated modal screening techniques and intelligent optimization algorithms based on this framework to further enhance design efficiency and performance.
